# Efficient Modeling and Active Learning Discovery of Biological Responses

**DOI:** 10.1371/journal.pone.0083996

**Published:** 2013-12-17

**Authors:** Armaghan W. Naik, Joshua D. Kangas, Christopher J. Langmead, Robert F. Murphy

**Affiliations:** 1 Lane Center for Computational Biology, Carnegie Mellon University, Pittsburgh, Pennsylvania, United States of America; 2 Departments of Biological Sciences, Biomedical Engineering and Machine Learning, Carnegie Mellon University, Pittsburgh, Pennsylvania, United States of America; 3 Freiburg Institute for Advanced Studies and Faculty of Biology, Albert Ludwig University of Freiburg, Freiburg, Germany; University of Catania, Italy

## Abstract

High throughput and high content screening involve determination of the effect of many compounds on a given target. As currently practiced, screening for each new target typically makes little use of information from screens of prior targets. Further, choices of compounds to advance to drug development are made without significant screening against off-target effects. The overall drug development process could be made more effective, as well as less expensive and time consuming, if potential effects of all compounds on all possible targets could be considered, yet the cost of such full experimentation would be prohibitive. In this paper, we describe a potential solution: probabilistic models that can be used to predict results for unmeasured combinations, and active learning algorithms for efficiently selecting which experiments to perform in order to build those models and determining when to stop. Using simulated and experimental data, we show that our approaches can produce powerful predictive models without exhaustive experimentation and can learn them much faster than by selecting experiments at random.

## Introduction

It is increasingly accepted that the study of biology requires a paradigm shift from a reductionist framework to a complex systems approach [1-3]. Reductionist frameworks implicitly assume that the object of study is comprised of a finite set of subsystems, each functionally and essentially physically distinct. In this case there is a reasonable upper bound for the total number of experiments necessary to characterize the whole, one experiment per component per subsystem. For complex systems the upper bound on the total number of experiments is the number of ways in which the components can be taken in combinations up to some maximum number per experiment (ten thousand components even taken only five at a time would require over 10^17^ experiments). 

This problem is manifest when trying to determine the effects of potential drugs on complex systems, since drugs with desired effects often have undesired side effects. It has been argued that these constitute the greatest component of risk in drug development since unforeseen deleterious behaviors are costly to correct [4,5]. The only way to be sure that a drug does not have side effects is to measure its effect in assays for all potential targets. Since explicit characterization in this manner is infeasible, approaches that do not require exhaustive experimentation need to be considered [6]. To do this, we must assume some structure or correlations exist within the complete data, and that predictive models can be used to capture them and guide future experimentation. Algorithms for this type of problem are termed Active Learning in the machine learning literature [7-10]. There have been limited applications of these methods to biological problems [11-15], but none in the context of multi-target, multi-drug analysis. Furthermore, the methods we present here are equally applicable to more general conditions than just drugs. In this paper, we show in extensive computational experiments that a combination of a structure learning method and active learning can achieve high accuracy of prediction of condition-specific effects on targets with significantly fewer experiments than a random learner, in many cases with perfect accuracy without exhaustive experimentation. The experiments were done with both synthetic and experimental data. Further, we provide a method for learning when to stop experimentation, a critical step for practical use of active learning. 

## Materials and Methods

### Definitions

**Figure 1:**
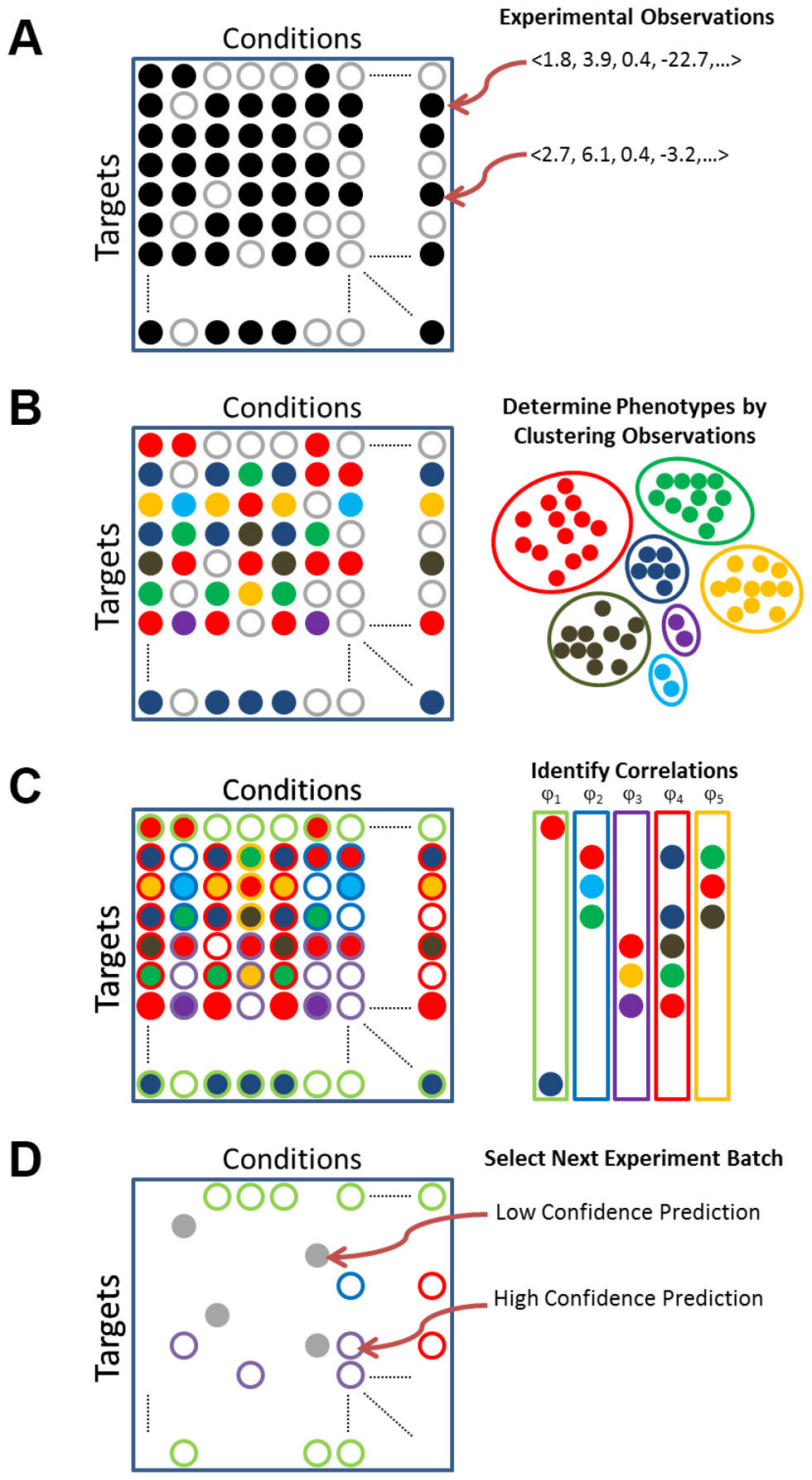
Active Learning Process. (A) An experiment is a combination of a target and a condition; observed experiments (filled circles) associate a target and condition with a vector encoding an experiment result. (B) Phenotypes (filled colored circles) are identified by cluster analysis of the experiment results. (C) From the arrangement of phenotypes across targets and conditions, a small set of correlations ϕ (distributions of phenotypes across targets) are identified which are then used to impute unobserved experiments. (D) A batch of experiments (filled grey circles) is selected based in part on predictions (outlined colored circles) from the identified correlations. The process (B-D) is repeated until a desired goal is met.

We consider a general problem consisting of finite sets of *targets* and conditions, combinations of which define an *experiment*, whose outcome is an *experimental result* (Figure 1). This is expressed as a categorical *phenotype*, and we are interested in knowing the phenotype for all possible experiments.

The inputs to the learning procedures considered here are a set of targets *T*, discrete conditions *C* and a procedure *F* which is used to form phenotypes from a space of observations *O*; *T* and *C* are fixed and finite. Observations arise by performing *experiments* taken from *T*x*C* (the *experiment space*). Observations are interpreted by *F* to produce *categorical phenotypes* F(O). Collectively, these define the *experiment result space* Ω=*T*x*C*x*F*(*O*); for convenience we also define a function *E* which returns the experiment of an experiment result: *E*(ω) = (*t*,*c*) when ω=(*t*,*c*,o).

The learners considered here do not initially assume that targets may be directly compared among themselves, nor that conditions may be directly compared among themselves. This allows us to consider potentially complicated experiment spaces. For instance, conditions may consist of addition of drugs, knockdown of gene expression, or changes in temperature – it is not clear how to directly compare (or express similarity between) temperature changes to drugs or drugs to gene knockdowns. Likewise, the targets may also be heterogeneous: some of the targets may be proteins, some may be RNAs and again it is not clear how to directly compare these. The phenotypes F(O) are therefore the sole basis of comparison: two experiments (*t*_1_,c_1_) and (*t*_2_, *c*_2_) are considered similar if they have the same phenotype. Various ways of extending this concept produces a way of measuring similarity of two targets across different conditions or vice versa.

The *learning process* constructs a sequence of predictive models over *E*(Ω) by iteratively performing *batches* of experiments; each step in the sequence is called a *round* of experimentation. We consider the case where experiments are acquired in batches of fixed size *S*; this models the case where it is cost-effective to perform several experiments at a time such as for high-throughput technologies. Each batch of experiments is disjoint to experiments already observed. The sequence of models progressively identifies nested subsets of Ω (and *E*(Ω)); after *n* rounds of experiments the collected data are Σ_n_ ⊆ Ω.

At each round the *structure learning problem* is to identify a predictive model *M*_n_ (*M*_n_[Σ_n_]). This may be used to propose a next batch of experiments *B*_n+1_ ⊆ *E*(Ω)*\E*(Σ_n_). Active learning strategies choose experiments based on observed data: *B*_n+1_|Σ_n_^~^ *f*(Σ_n_) for some function *f*, whereas a random learner ignores the dependence and uniformly samples *S* experiments from the remainder: *B*_n+1_|Σ_n_^~^ Uniform[*E*(Ω)*\E*(Σ_n_)].

## Structure Learning

We introduce a model class which assumes that observations *O* are distributed in condition-specific manners. That is, we will estimate a set of distributions Φ, the size of which is re-estimated each round. Each distribution ϕ is a function from a subset of the targets *T* (called its “support”) to the set of phenotypes F(O); for targets not in the support of a distribution, no phenotype is associated. For each condition *c*, there is at least one distribution that can make predictions for some of the targets. Informally, since several conditions can be associated with the same distributions, these *correlations* describe mutual predictions from one target-phenotype pair to another across conditions. From these we can build an asymmetric model of the distribution **P**[F(O) | (*t*,*c*)]. 

The conditional independence structure is encoded by a *valuation* Γ which indicates which distributions each experiment (*t*,*c*)∈*E*(Ω) depends on. For convenience, we assume an indexing of the distributions. Formally, a valuation Γ :*T x C* → 2^[|Φ|]^ maps an experiment to a set of indices over the distributions. Independence of two experiments *e*_1_,e_2_∈*E*(Ω) is expressed as disjoint valuations, **P**[*e*_1_] ⊥ **P**[e_2_] ⇒ Γ(*e*_1_) ∩ Γ(e_2_) =∅; informally this means that these two experiments were estimated to have their phenotypes by unrelated causes. A *choice operator* ε resolves cases where an unobserved (predicted) experiment has multiple valuations (|Γ(*e*)|>1) to form coherent predictions; different ε lead to different generalizations.

Choices for these form a model *M* = (Φ,Γ,ε). Predictions for an observed experiment ω=(*t*,*c*,o) in Σ are produced through Γ:

$$P[F(O)|E(\omega ),M]=\phi_{\Gamma (t,c)}[t]=F(o)$$

In words, the predicted phenotype of an observed experiment is such that the valuation of the experiment is a distribution that maps the target to the observed phenotype. Estimates for observed data do not depend on ε. Predictions for unobserved (*t*,*c*) ∈ *E*(Ω)\*E*(Σ) are also constructed over Φ and Γ. To do this, for every condition we identify the distributions that could be used to make predictions for unobserved targets in that condition. These sets Γ^(c)^ are given by the common refinement

$$\underset{\left(t,c)\in E(\Sigma \right)}{\cup }\Gamma (t,c)$$

Since the correlations in Γ^(c)^ may make different phenotype predictions for the same target, the choice operator will pick one of them. Taken together, given a model *M* = (Φ,Γ,ε), predictions (when they exist) are defined as

$$P[F(O)|(t,c),M]=\{\begin{array}{cc} \phi_{i}[t] & \text{if }i=\Gamma (t,c)\text{ and }(t,c)\in E(\Sigma )\\ \phi_{i}[t] & \text{if }i=\varepsilon (\Gamma^{\left(c\right)})\text{ and }(t,c)\notin E(\Sigma ) \end{array}$$

These predictions may be augmented by various data imputation methods (described below). In their absence, we choose ε to be the function such that we predict the most common correlation for each target to make a phenotype prediction.

We considered two methods, a “Greedy Merge” and a Quantified Boolean Formula Satisfaction (QBF/SAT) [16] based estimation procedure termed “B-Clustering.” 

### Greedy Merge Structure Learning

Greedy Merge produces Φ and Γ from data and a clustering of observations by iteratively combining condition-specific distributions under the assumption that some of the conditions affect all targets in the same ways. These are determined by iteratively computing model estimates M_z_ = (Φ_z_, Γ_z_, ε) which are monotone decreasing in the size of Φ. We considered two variants, one variant considered performs the first two steps below and the second variant, Greedy Merge which is used throughout our work, performs all three steps below.

#### Initialization

Let M_0_ = (Φ_0_, Γ_0_, ε). Associate a ϕ_c_ with every *c* ∈ C such that for all observed (*t*,*c*,o) ∈ Σ, ϕ_c_[*t*] = F(O). Set Φ_0_ to be the set of all ϕ_c_, and Γ_0_(*t*,*c*) = *c*. This produces an initial model estimate where observed experiments are assumed conditionally independent if they differ in condition. 

#### Merge Overlapping

To produce M_z+1_ from M_z_ = (Φ_z_, Γ_z_, ε), arbitrarily choose two different ϕ_i_ ,ϕ_j_ ∈ Φ_z_ such that their supports overlap and in the overlap predictions do not differ (ϕ_i_[t] = ϕ_j_[t] for *t* in the common support). Set fresh ϕ_z_ = ϕ_i_ ∪ ϕ_j_. Replace ϕ_i_, ϕ_j_ with ϕ_z_ to make a new Φ_z+1_. Likewise, update references to *i* and *j* in Γ with *z*. This step is iteratively applied. At termination there are no more overlapping ϕ_i_,ϕ_j_ to merge and so *M*_z_ distinguishes between two experiments *e*_1_, e_2_ if the distributions they are assigned to in Γ differ in any target’s phenotype. *M*_z_ may produce identical predictions for some target *t* across two conditions c_1_, *c*_2_ (**P**[F(O)|(*t,c*_*1*_)] = **P**[F(O)| (*t,c*_*2*_)]) but treat them as conditionally independent events (Γ(*t*,c_1_) ∩ Γ(*t*,*c*_2_) = ∅) if there is some other *t*' where **P**[F(O)| (*t*',c_1_)] ≠ **P**[F(O)| (*t*',*c*_2_)]. 

#### Merge Nonconflicting

This step is similar to Merge Overlapping, but the requirement that two distributions have common support is removed and any two nonconflicting distributions can be merged.

### B-Clustering

An alternative procedure would be to define properties that are believed to describe “good” models of the data, and then use an efficient search procedure (a satisfiability solver) to find examples of those models. This is most helpful when it is unclear how to construct an algorithm that directly estimates models which will satisfy the desired properties. We considered the use of Quantified Boolean Formula (QBF/SAT) methods built using the MiniSat solver [17] to identify a model subject to constraints defining an optimum. In this framework, each observed target and phenotype pair is associated with an index of a distribution. This implicitly defines distributions (which map targets to phenotypes) as the collection of target and phenotype pairs with the same index. To do this, each unique observed target and phenotype pair (*t*,*F*(*o*)) is associated with a vector of literals ν_t,o_ which encodes in two's complement the index of a distribution in Φ (e.g. a binary encoding of a natural number). Legal assignments of each of these literals to true or false will define the distributions. The set of legal assignments is constrained by introducing logical formulas which encode different criteria. 

An example criterion is to constrain the choice of model such that each (*t,F*(*o*)) is described by exactly one distribution ϕ_I_; ensures that each distribution predicts at most one phenotype per target, and that all occurrences of a particular target and phenotype pair must have a common cause. This is encoded in a per-target constraint SingleOwner(t) which asserts that for the set Ξ[t] of all (*t*, *F*(*o*)) with the same target, their distribution indices ν_t,o_ must be different.

$$SingleOwner(t)=\wedge \left\{v_{t,o}\neq v_{t,o'}\text{ if }\{(t,o),(t,o')\}\in \left(\begin{array}{c} \Xi [t]\\ 2 \end{array}\right)\right\}$$

Another criterion (Coobserved(*t*,o)) is that for each distribution ϕ_I_, each pair of distinct targets *t*,*t*’ in the support is coobserved at least once in some condition *c*. That is, we disallow distributions which make predictions that are totally unsupported by mutual observations. Let β(*t,F*(*o*)) be the set of conditions that a pair (*t,F*(*o*)) was observed in.

$$Coobserved(t,o)=\begin{array}{c} \left(\vee \left\{v_{t,o}\neq v_{t',o'}\text{ for all }(t',o').\left|\beta (t,o)\cap \beta (t,o)\right|>0\text{ and }t\neq t'\right\}\right)\vee \\ \left(\wedge \left\{v_{t,o}\neq v_{t',o'}\text{ for all }(t',o')\neq (t,o)\right\}\right) \end{array}$$

A third criterion restricts the valuations of each condition (Γ^(c)^) to be disjoint, so that predictions of unobserved targets for each condition are always unique.

$$Noncontradiction((t,o),(t',o'))=v_{t,o}\neq v_{t',o'}\Rightarrow \begin{array}{c} \left(\wedge \left\{v_{x}\neq v_{t,o}\text{ if }x\in \Xi [t]\right\}\right)\wedge \\ \left(\wedge \left\{v_{x}\neq v_{t',o'}\text{ if }x\in \Xi [t']\right\}\right) \end{array}$$

Other conditions may be applied. The model estimate chosen is found by identifying the least number of distributions *N* such that the SAT solver finds a solution where all of the above hold:

$$\underset{N}{argmin}\exists N.\begin{array}{c} \left(\underset{t}{\wedge }\text{SingleOwner}(t)\right)\wedge \left(\underset{(t,o)\in \Xi }{\wedge }Coobserved(t,o)\right)\wedge \\ \left(\underset{\left|\beta (x)\cap \beta (y)>0\right|}{\wedge }Noncontradiction(x,y)\right)\wedge \left(\underset{(t,o)\in \Xi }{\wedge }v_{t,o}<N\right) \end{array}$$

### Imputation as Model Augmentation

Ordinarily data or model imputation methods attempt to correct situations where most data are available and only a very small set are missing at random. In these situations, it may be reasonable to impute missing data by marginal estimates. Our learning problem is diametric: most of the data are missing and not at random. We therefore chose two alternate imputation rules to augment the model. For each we modify ε to either be the unique imputed phenotype (if it exists) for some (*t*,*c*) or the imputation arising from the most common correlation for that *t*. However, we keep all possible imputations for each (*t*,*c*) in a relation *I* which maps from *T*x*C* to subsets of the phenotypes F(O).

### Target Equivalence Estimation

A simple imputation procedure estimates equivalence classes of targets as measured by common or similar observations. If two targets agree in their observations everywhere that they are coobserved then we may reduce the model by associating the predictions of one with the other, possibly leading to a larger set of concrete predictions for both.

### Three-Point Imputation

Deductive reasoning produces other structural assumptions. We can interpret each distribution ϕ∈Φ as an assertion that for any two distinct targets *t*,*t*' in its support, whenever we observe in a condition *c* that one target *t* had phenotype ϕ[t] we may predict that an unobserved experiment (*t*',*c*) has phenotype ϕ[*t*']. If we iterate these predictions by assuming the largest set possible of them, we can potentially make many more predictions than are immediately justified by the model. Formally, for each distribution ϕ_i_ we form the relation

$$R[\varphi_{i}](t,c)⇐\exists t'\text{. }\Gamma (t',c)\text{ and }\exists c'\text{. }\Gamma (t',c')\text{ and }\Gamma (t,c')$$

An experiment (*t*,*c*) is in R[ϕ_i_] if there was a way to obtain pairwise target predictions of ϕ_i_ as described above from some other condition *c*'. We write the transitive closure of R[ϕ_i_] as cl R[ϕ_i_]; this relation captures the logical extension of ϕ_i_ to as many (*t*,*c*)∈*E*(Ω) as possible by iterating until no new experiments are added. These are computed for each distribution ϕ separately. We interpret the case where (*t*,*c*)∈**cl** R[ϕ] as weak predictions: “the phenotype of experiment (*t*,*c*) *might* be ϕ[t].” Since an unobserved experiment (*t*,*c*) can be in the closure of R for different distributions, it is sometimes the case that there are multiple and distinct weak predictions for that experiment. That is, if (*t*,*c*)∈ cl R[ϕ_1_] and (*t*,*c*)∈ cl R[ϕ_2_] it can be the case that ϕ_1_[t]≠ϕ_2_[t]. The set of unobserved experiments that have multiple weak predictions are where the model may be considered *concretely uncertain* as opposed to simply latent.

### Active Learner

A batch learner sequentially proposes experiments for observation given observed data. At batch step *n*, given data Σ_n_, the following are provided: model *M* = *M*_n_[Σ] = (Φ,Γ,ε), the collection of all possible imputations *I* and the model reductions *R* ⊆ 2^T^ used to form *I*. The goal is to balance choosing experiments amongst all those with imputations in *I*, and all possible refutations of identified correlations, taking into account any symmetry relationships induced by *R* and their refutations. Each unobserved experiment is given a rank reflecting the number of distinct imputed observations and through R, I and Φ forms a set system. The next batch *B*_n+1_ is computed as a weighted *S*-hitting set so as to minimize the number of experiments expected to be imputable from each other and to refute the greatest number of assumed conditional independences. 

### Ranking Experiments and Symmetry Breaking

We partition *E*(*U*_n_) into disjoint subsets, *U* ^I^, *U* ^\I^ where U^I^ = *E*(*U*_n_)∪*E*(*I*) and U^\I^ is the remainder (slightly abusing notation for *E*). We form a lookup *R* which returns all the targets which are in the same model reduction equivalence class; if one was not estimated, then *R* is just the identity map. Let C_u_ be those *c*∈*C* with no observations in Σ_n_; this set is usually empty after learner initialization. A weak association on *C* x 2^C^ is introduced in the following manner: for each *c*, let *Q*(*c*) be the relation that identifies those *c*'≠*c* whose model predictions are equal for some *t*∈*T*. *Q*(*c*) need not be symmetric and is always irreflexive. *Q* is used to break symmetry through *R* in batch selection by the relation *W*, which identifies those unobserved (*t*,*c*) with any (*t*',*c*') such that *c* is weakly associated to *c*' (*c*R*c*') and the model predictions differ (**P**[F(O)|(*t*,*c*)]≠ **P**[F(O)| (*t*',*c*')]). In words, *W* marks those experiments which have shown any variation amongst similar conditions.

Given the above, a rank *z*(*t*,*c*) is computed over *E*(*U*_n_). For each (*t*,*c*), define the pre-rank *z*’ to be the number of imputations for (*t*,*c*) that have different phenotype predictions:*z*'(*t*,*c*)=|{*φ*_*i*_[*t*] for (*t*,*c*,*φ*)∈*I*}|. Rank is defined as:

$$z(t,c)=\{\begin{array}{cc} W(t,c)+1 & \text{if }z'(t,c)=1\\ W(t,c)+z'(t,c)+3 & \text{otherwise} \end{array}$$

Notice that this ranks all elements in U^\I^ over experiments with a single concrete imputation. Informally this chooses experiments that have many possible imputations, and then those with no imputations and only then consider choosing experiments that have single imputations. 

### Batch Selection

From these ranks, a weighted *S*-hitting set is computed as *B*_n+1_ so as to minimize the number of experiments expected to be imputable from each other through *R* and Γ^(c)^. This is done greedily, starting from the set of greatest rank, choosing an unobserved experiment uniformly at random, and then (temporarily) eliminating from consideration all those experiments reachable through *R* and then selecting a next experiment from the greatest nonempty rank set by repeating. If *S* many elements have not been selected, then the temporarily removed experiments are placed back into consideration and the selection process is again applied; this case generally only occurs when the apparent uniqueness of the data is very low. 

### Learner Initialization

The learning process initializes from an empty Σ_0_ to request $i=\left\lceil \frac{\left|T\right|+max\left(\left|T\right|,\left|C\right|\right)}{\left|S\right|}\right\rceil$many batches of experiments. These *S* x *i* many experiments will cover two sample sets. The first is all targets under the unperturbed condition. The remaining initializing experiments consists of a scoreboard of max(|*T*|,|*C*|) points chosen such that each target and each condition is sampled at least once, with the possibility of padding points chosen at random to fill a complete batch *B*_i_. This starting choice for Σ_i_ allows Target Equivalence Estimation to produce a maximal (but not necessarily accurate) upper bound equivalence reduction and observes every target at least twice which provides a reasonable initial minimum bound estimate of the number and partial identity of correlations.

### Parameterization of Experiment Problem Space

A description of experimental spaces with an equal number *N* of targets *T* and conditions *C* can be parameterized in three terms θ=(m, λ_r_, λ_u_) as follows. For convenience, fix an ordering of *T* and *C* each over [N] with condition *c*=1 as the unperturbed condition. Influenced conditions *c*∈2..*N* are perturbations from the unperturbed condition. Let *m* be the size of F(O). When the observation for a particular *t* differs in condition *c*≠1 from condition *c*=1 we say that the experiment was *responsive*; let λ_r_ be the expected fraction of targets that are responsive. Different *t* may have identical response across *C* and likewise different *c* may similarly perturb *T*; let λ_u_ be the expected fraction of each of *T*,*C* that are unique up to equivalence through phenotypes. λ_r_ and λ_u_ are therefore rate parameters for a truncated Poisson distribution.

A choice of θ generates data Ω = Ω [θ] by the following process. Let *n*_T_, *n*_C_ be the number of underlying (to be replicated) targets and conditions respectively, *n*_*T*_=⌈(*N*−1)*λ*_*u*_+1⌉and similarly for *n*_C_. For each unperturbed experiment (*t*,1) sample uniformly with replacement from [m]. Sample *n*_C_-1 times from the truncated Poisson distribution to determine the number of responses per responsive condition. For each condition c ∈ 2..*n*_C_ choose *d*_c_ many indices in [*n*_T_]; observations for these indices are set distinct from the unperturbed condition. The data are completed by sampling with replacement from [*n*_T_] to fill out the N - *n*_T_ many replicated *T*, and similarly for *C*.

### Predicted Accuracy Score Regression and Stopping Rule Construction

To characterize a model learned at a particular batch, we measured several features on both that model and on differences between that model and the model learned at the previous batch. All of these features are based on data available to the model; in particular, the parameterization of data used was not included. These features fell into several broad categories.

The first set of features measured simple counts: (1) the current batch number, (2) the number of distributions in the model, (3) the number of unique phenotypes observed, (4) the number of experiments whose (predicted) phenotype is in agreement between the previous model and the current model and (5) the number of experimental conditions that differ within a target.

The next set of features measures aspects of the model as a Markov hypergraph system: (6) the minimum fraction of each *current* distribution that was observed in the *previous batch* a particular condition, (7) the maximum fraction as above (6), (8) the maximum of the fraction of *current* imputations or distributions that the *previous batch* covered (e.g. how good an ε-approximation the last model was to the current model) (9), the difference of the average number of each phenotype observed between the *previous* and *current* models and (10) the size of the maximal matching of distributions between the *previous* and *current* models.

These features were combined with their pairwise products and z-scored and formed the design matrix for regression. The dependent variable was the measured accuracy was adjusted by subtracting the percentage of the population observed per-batch; this essentially removes the expected fraction of accuracy one would expect at random. The design matrix was regressed in logistic lasso [18] against the adjusted measured accuracy; the choice of regularization constant was determined by minimizing 10-fold cross validation (folds formed over the whole of the data). Loadings were computed by ordinary least squares fit using the nonzero features identified by lasso regression, and used to produce predicted accuracy scores from the design matrix. The resulting scores were then re-adjusted by adding back in the percentage of population observed per-batch and normalized so that the maximum was 1.0 instead of ~1.1.

### Gene Expression Analysis

Normalized gene expression data were taken from the Connectivity Map [19,20] dataset (available at http://lincscloud.org as of time of writing). The dataset consists of gene expression profiles in 48 cell lines under treatment by 280 drugs. We identified a completely observed submatrix of 50 highly drug-responsive genes (targets), 280 drugs (conditions) and formed phenotypes of the measured gene expressions across the 48 cell lines by *k*-means clustering. To identify the 50 genes, expression levels were z-scored per-gene and ranked by variance explained by 280 treatments (variance of gene expression levels conditioned on drug). The 50 genes most varying according to treatment were chosen so the resulting dataset was not trivial (i.e. there would likely be more than one phenotype) and to limit computational requirements for simulation. A (280x50, 48)-matrix of observations across cell lines was formed with averages of technical replicates and clustered with varying *k-*means; for each *k* the model that minimized reconstruction error from 200 seeds was used. For each of these, a (280, 50)-matrix was formed from the phenotypes for the simulations to query.

### Availability

Scripts for setting up the simulations and generating figures from the results are available from http://murphylab.web.cmu.edu/software. Active learning software will be made available for non-commercial use upon request.

## Results

### Learning Problem

As described in the Methods, we consider a general problem consisting of learning a model for the effects of different *conditions* upon different *targets* (the combination of which define an *experiment*) (Figure 1a). The result of each experiment is expressed as a categorical *phenotype*. Given some initial data, either in the form of phenotypes or other measurements from which we can obtain phenotypes (Figure 1b), we learn correlations between the behaviors of targets and conditions that allow us to make predictions for unobserved experiments (Figure 1c). We then construct a *batch* of experiments to observe next in order to improve the model (Figure 1d).

For this task, we considered different possible *learning processes*, each comprised of (i) a *probabilistic model*, (ii) a *structure learning* method for the model, (iii) a choice of data *imputation* methods and (iv) a choice of *active* or *random learning strategy* along with (v) a *stopping rule* which gives an estimate for when a ‘good enough’ model has been learned (Methods).

### Model Selection

In order to test the ability of the models described above to support active learning, we performed computational experiments for several model designs. For these simulations, we generated datasets consisting of *m* phenotypes for a set of targets and conditions. Each target was assigned a base (unperturbed) phenotype; the probability that a target would change phenotype for other conditions was given by a parameter λ_r_ (“responsiveness”). The extent to which targets showed the same responses across all conditions, and the extent to which conditions had the same effect on all targets, was controlled by a parameter λ_u_ (“uniqueness”). For illustration, λ_u_=1 would correspond to all targets and conditions showing a unique combination of phenotypes, and λ_u_=0.1 would correspond to an average of 10% of targets and conditions showing the same combination.

**Figure 2:**
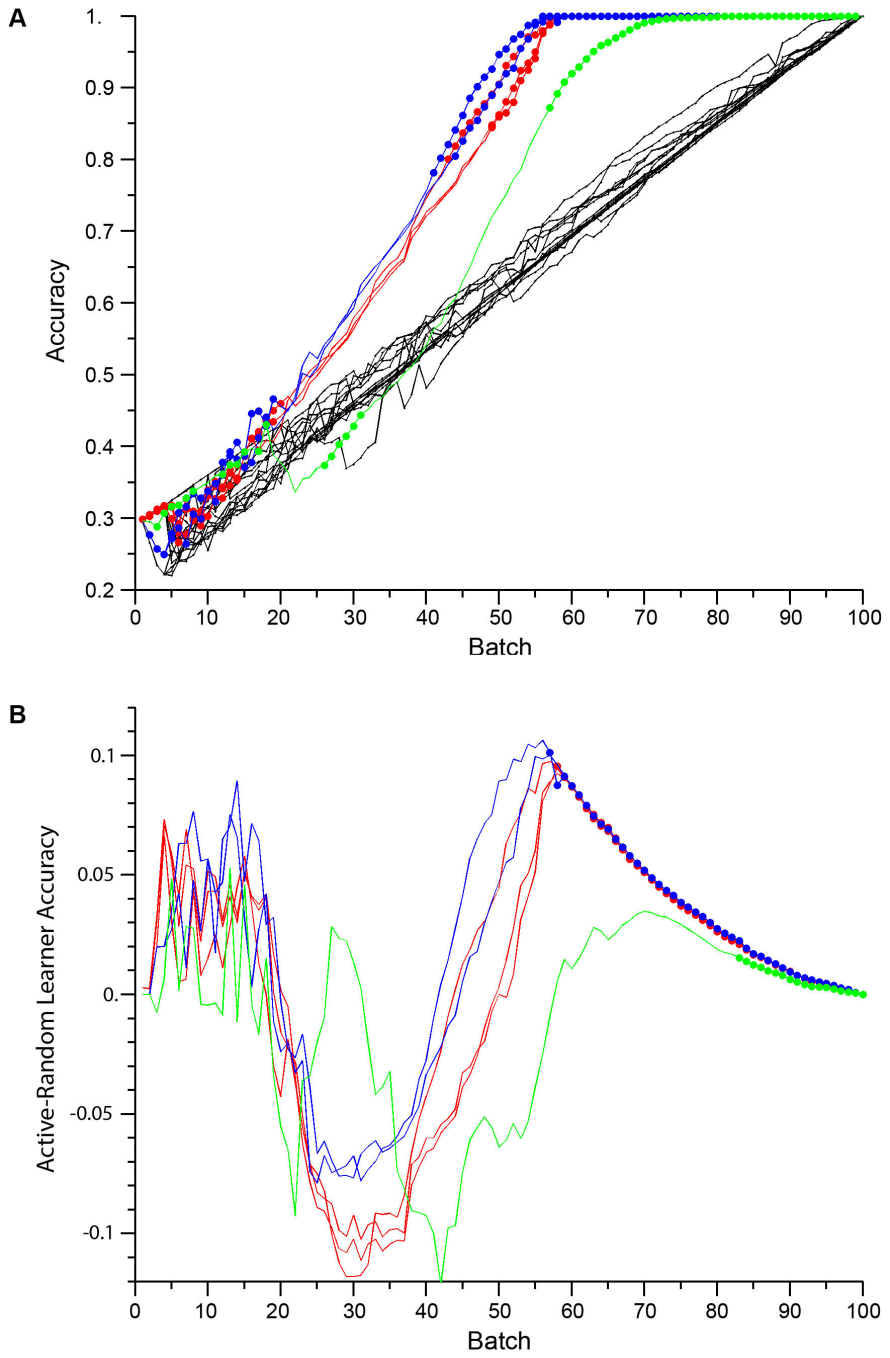
Learning performance dependence on model design: structure learning and imputation rule choice. (A) Each model design was evaluated with both active and random learners on two simulated 100 target x 100 condition datasets, each having eight phenotypes, 80% responsiveness and 40% uniqueness. For each model design the best average accuracy for either the active or random learner is plotted at each batch. For six cases displaying superlinear performance, structure learning methods are indicated in color, with different design variations plotted as separate lines and with filled circles to indicate batches where the active learner had higher accuracy: Greedy Merge (blue), a ‘strict’ variation of Greedy Merge (red), and B-Clustering (green, one design). These each had both Target Equivalence Class and Three-Point Imputation rules. (B) The difference in random and active learner accuracies for the superlinear model designs with structure learning method plotted by color as above; filled circles at tails indicate that the active learner had reached 100% accuracy.

We performed computational experiments for several model designs, each consisting of a choice between two structure learning methods (*Greedy Merge* and *B-Clustering*) with predictions augmented with one of four combinations of imputations. The simulations were evaluated for 100 targets and 100 conditions with parameterization θ=(*m*=8, λ_r_=80%, λ_u_=40%) with a fixed batch size of 100 (Methods). At each batch the best accuracy for either the random or active learning strategy was chosen as an indication of how well that design can perform. These are displayed in Figure 2A. Most model designs showed linear increase in accuracy with batches as would be expected for a model-free random sampler. Only five model designs showed learning that was superlinear. The batch-wise difference between active and random learning accuracies for these five designs are shown in Figure 2B. Different designs show peaks in improvement over random after different numbers of batches have been observed.

### Model Performance

**Figure 3:**
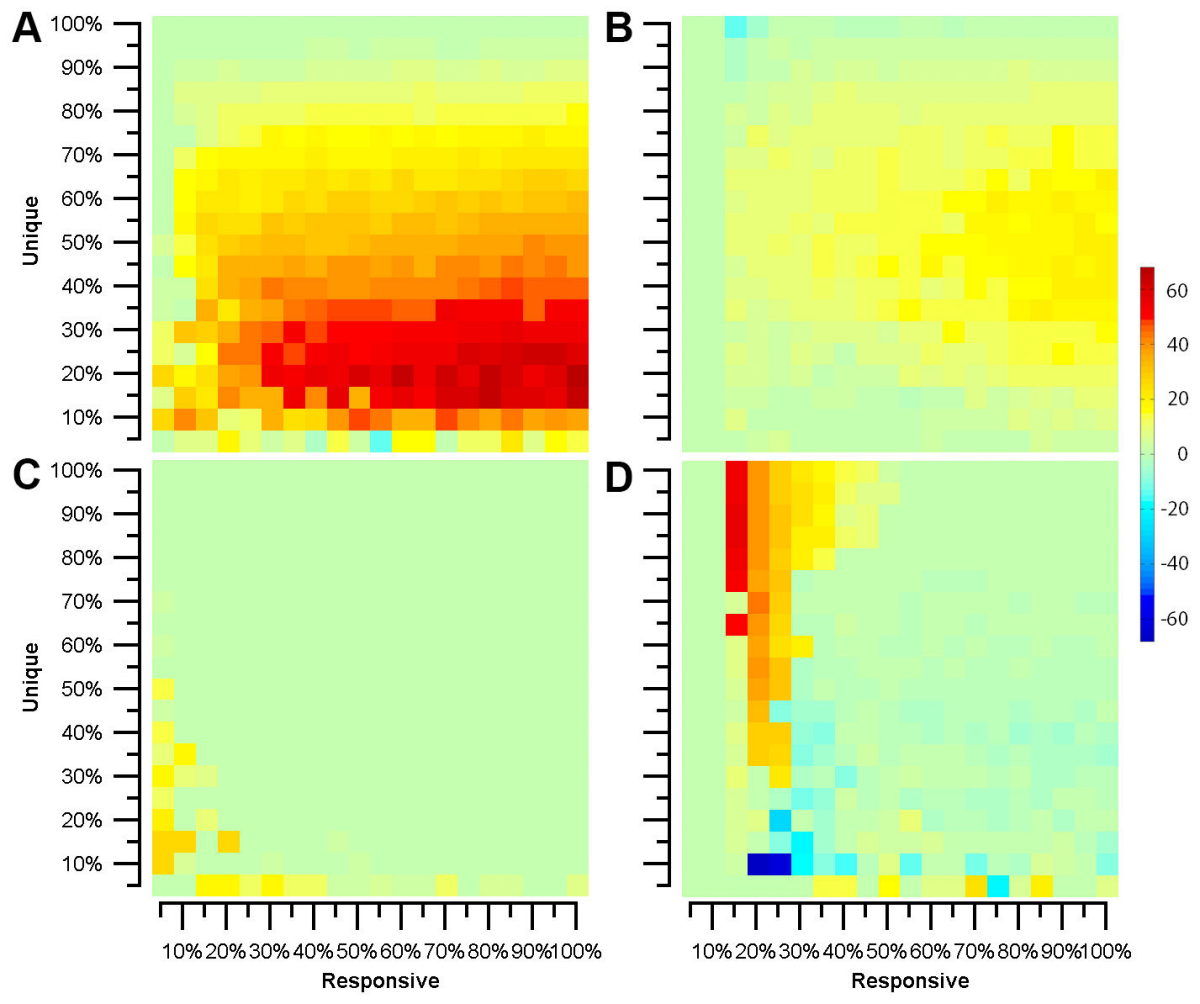
Active learning performance for different model designs. Performance was measured as the difference in the number of batches to achieve (A,B) 100% or (C,D) 90% accuracy between active and random learning. (A,C) Greedy Merge, (B,D) B-Clustering. Warmer colors indicate greater experiment savings with an active learner.

We then evaluated the performance of active and random learning methods for each of these model designs across a broad range of λ_r_ and λ_u_ for 32 phenotypes. We measured the difference in the number of batches required to achieve 100% predictive accuracy between active and random learning methods. As Figure 3A indicates, our active learning strategy with Greedy Merge structure learning achieved 100% predictive accuracy more rapidly than random learning over the majority of the sampled range of λ_u_ and λ_r_, with qualitatively similar behavior for 90% accuracy (Figure 3B). The improvement is much less for B-Clustering (Figure 3C,D). However, as discussed below, there are cases where each method dramatically outperforms random sampling.

**Figure 4:**
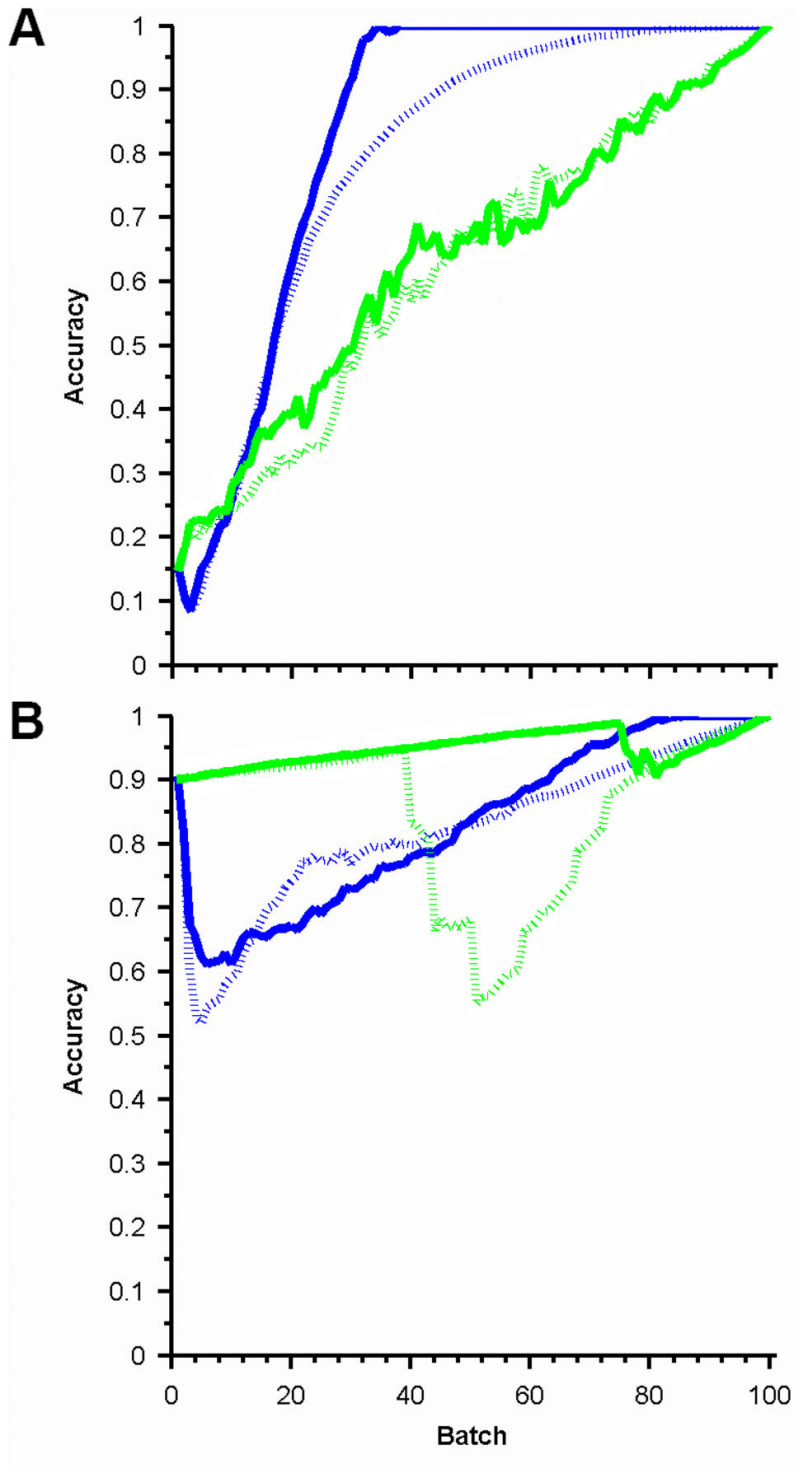
Example learning curves. Mean learning rates for active (solid) and random (dashed) learners across structure learning methods, Full Greedy Merge (blue) and B-Clustering (green). Data from experiments in Figure 3 for (A) (λ_r_=90%, λ_u_=25%); (B) (λ_r_=10%, λ_u_=70%).

Figure 4 shows example learning curves for specific combinations of λ_u_ and λ_r_. The most striking conclusion (echoing Figure 2) is that the models learn much more rapidly than random sampling. Figure 4A shows a case that with a high λ_r_ and low λ_u_. The initial models are poor in these cases as predictions from the unperturbed condition do not generalize well, but rapidly improve as correlations are learned, generalized and used to identify likely responsive experiments. The combination of the Greedy Merge model with active learning gives a perfect accuracy after only about 30% of the data have been sampled. By contrast, the “needle in the haystack” case in Figure 4B (small λ_r_ and large λ_u_) is initially predicted well by either learner with either structure learning method but further progress is slow and occasionally leads to poor models. Nonetheless, high accuracy is achieved before full sampling. Overall, while the efficacy of different active learning methods varies somewhat for different λ_u_ and λ_r_ values, the results of Figures 3 and 4 show a significant benefit in sampling with our active learners for the same number of batches as compared to a random learner in almost all cases (an important conclusion since λ_u_ and λ_r_ will not usually be known).

### Probability of Approximate Correctness

One potential problem with using active learning to perform only selected experiments is knowing when to stop. We therefore asked if it is possible for an experimenter to estimate the predictive accuracy of an actively learned model without completing all experiments. One way to do this would be to form a prediction of the accuracy of a model and a *confidence* that measures how likely the true accuracy (which the experimenter does not know) meets or exceeds the predicted accuracy.

We empirically evaluated this possibility for the Greedy Merge model by simulating a broad range of data with dimensions as before. These data were formed by randomly and uniformly sampling parameters in the cube (*m*=18..100, λ_r_=5..95%, λu=5..95%). For each of these, we measured *features* at every batch that described differences between the model learned at the previous and current batches. Features were limited to knowledge available to the learner at a particular batch and not reliant on unseen data, or on the parameters the data were drawn from. These features were then collected and regressed against the true model accuracy to produce a predicted accuracy score (Methods)*.*

**Figure 5:**
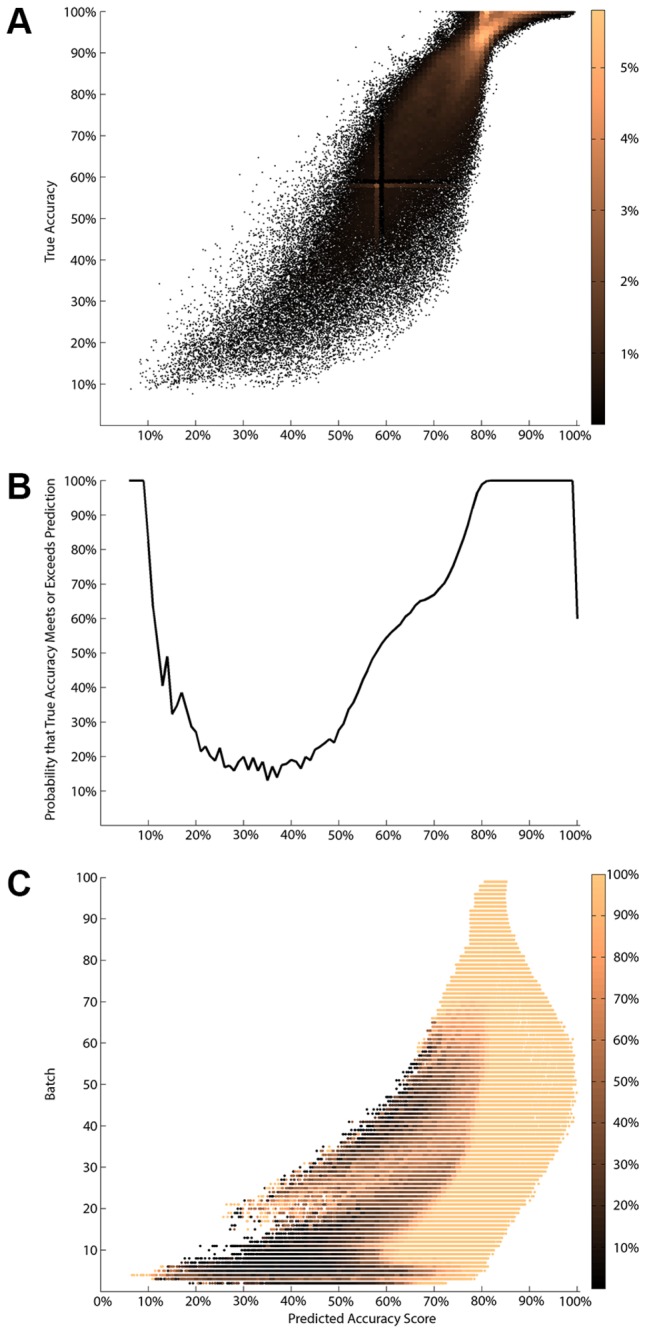
Probability of approximate correctness over a broad range of data. (A) The empirical density of the correspondence between the predicted accuracy score and the true (latent) accuracy; lighter colors indicate greater frequency. (B) Confidence (in units of probability) per level set of predicted accuracy score. (C) Per-batch and (1% binned) predicted accuracy score confidences; color indicates confidence (in units of probability).

The predicted accuracy score is in general a conservative estimate of accuracy, with the highest correspondences at higher true accuracies (Figure 5A). On the whole (Figure 5B) extremes in the true accuracy are identified with high confidence. A practitioner may then be confident that a model with a predicted accuracy score above ~80% is almost certainly at least that good. Furthermore the per-batch and predicted accuracy score confidences (Figure 5C) are conservative estimates everywhere. As an example, for a model acquired early in the learning process (batch 10) if we obtain a predicted accuracy score of 70%, we can be ~90% certain that the true model accuracy is in excess of 70%. Likewise, hard to learn cases are identified as such with low predicted accuracy scores or low confidence. With these a practitioner may choose a minimum target accuracy, or limit the total number of experiments performed, and still assert a quantitative bound on the accuracy of the model.

### Application: Learning the Effects of Drugs on Gene Expression Levels across Cell Lines

**Figure 6:**
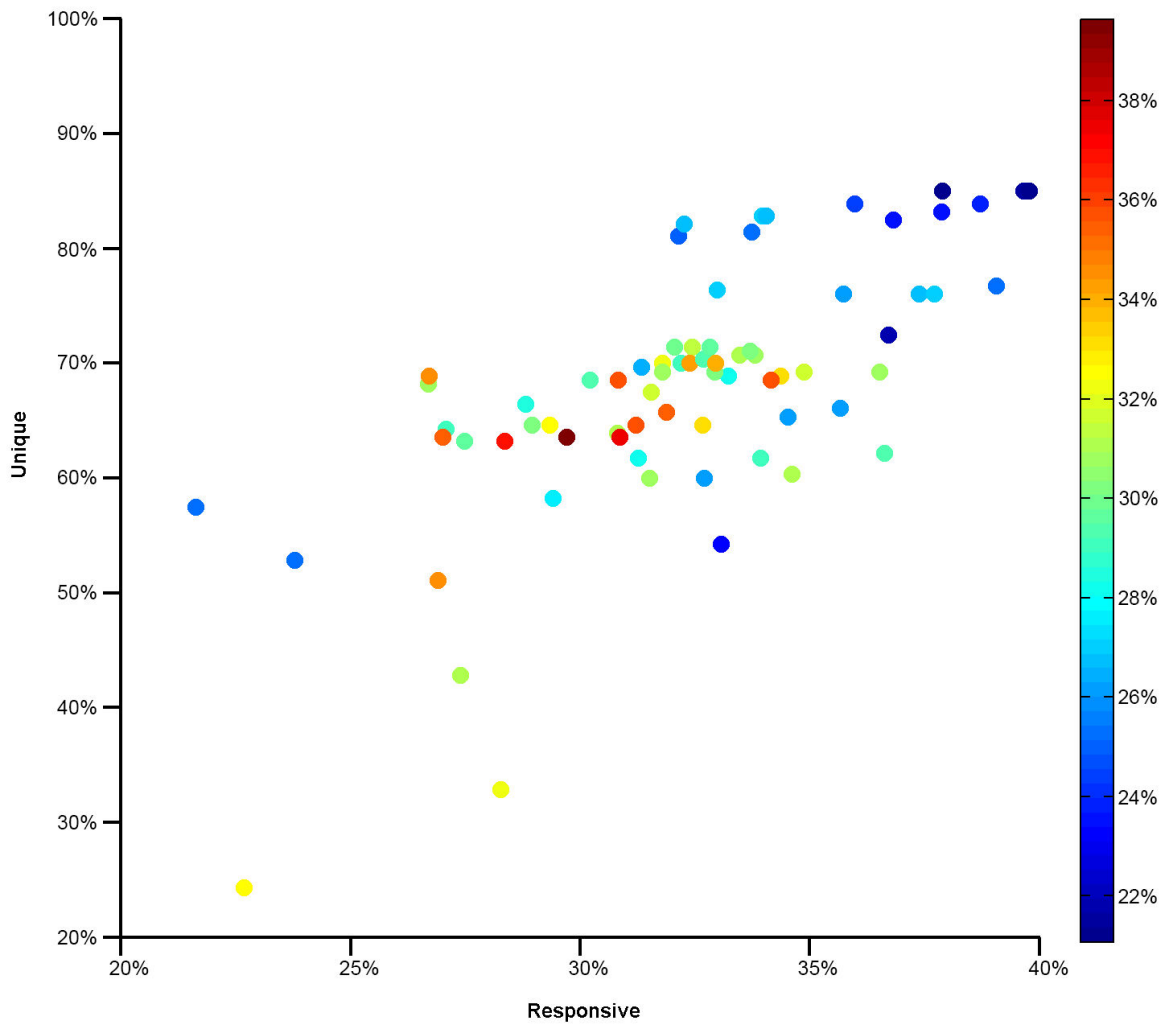
Learning the effects of drugs on gene expression levels across cell lines. Gene expression levels of the genes that varied most across drug treatments were used to form experimental observations across 48 cell lines. Each point represents a different number of phenotypes, varying from two (bottom left hand point) to 73 (upper right hand point). Warmer colors indicate greater experiment savings with an active learner.

In order to demonstrate the utility of this approach using experimental data rather than simulated data, we applied the Greedy Merge model to a dataset of gene expression profiles in 48 cell lines under treatment by 280 drugs. An unresolved issue is how to decompose these profiles into distinct phenotypes. To avoid justifying a specific choice, we considered a wide range of possible values (2.73) for the number *m* of distinct expression phenotypes and formed them by *k-*means clustering. For a given number of phenotypes, we can calculate the average λ_r_ and λ_u_. Figure 6 shows the improvement of Greedy Merge with Active learning over Random learning as a function of these average λ_r_ and λ_u_ values. Consistent with Figure 3, a 21%-40% reduction in the percent of experiment space required to achieve 95% accuracy was observed.

## Discussion

We have described a learning approach suitable for the study of large, complex systems where the constituents have unknown or incomparable relationships. We have developed and presented empirical characterization of a class of models that capture the structure which target-condition dependence exhibits, structure inference algorithms for the class of models that are suitable for sparse data and methods for imputing missing values based on the structure of the learned models. Importantly, since different targets may be part of very different biological mechanisms, and yet have correlated responses in various conditions, the models capture patterns in the phenotypes without assuming a causal structure among the targets. From these we have described and evaluated a batch active learner capable of sequentially proposing informative experiments. Our results show that it is possible to learn highly accurate models without exhaustive experimentation.

Critically, we have also shown that it is possible to produce an estimate of probable approximate correctness of the learning process without access to complete data. To the best of our knowledge, this is the first nontrivial active learner that (empirically) enjoys useful learning guarantees analogous to classical random sampling methods. This permits a decision about when an active learning process can safely be stopped.

An important application of this work will be to efficiently identify and model the dependencies of cellular targets upon potential drugs or drug cocktails; we are unaware of previous methods approaching the efficiencies reported here. Towards this, we were able to show that the expression levels of genes across diverse cell types under different drugs can form consistent patterns whose emergent structure can be accurately and rapidly learned. Interestingly, our results indicate that while it is possible to learn efficiently even for the binarized case (two phenotypes), there are may be even greater efficiencies when considering finer granularity of drug responses.

The learning problem here is similar to other well-studied problems. DNF formula learning [21] and multiarm bandit optimization [8] commonly consider categorical constituents and restrictions to equality comparisons. Furthermore, as with black-box optimization [22], we make very weak assumptions on the structure of the data and rely on nonparametric estimates. The tradeoff for weak data assumptions is that nonparametric methods are generally data biased predictors [23]. Close alternatives to our approach generally make parametric assumptions on the structure and topology of data. In particular, matrix completion [24,25] and similar regression-based methods are the natural extension of our models but require algebraic invariants on the marginal distributions of data [26,27]. We were motivated to explore the approaches presented here as we thought they would perform better in cases with sparse, not missing at random data that would be expected to be obtained from an active learning process. 

Our formulation of the target-compound problem intentionally ignores any prior information about similarities among targets and among compounds (i.e., since they are potentially inaccurate). However, in separate work we have demonstrated that including it with active learning can increase the learning rate (Kangas, Naik, Murphy, submitted). The availability of both types of methods will be important to future work in this area.
